# The *K*-mer antibiotic resistance gene variant analyzer (KARGVA)

**DOI:** 10.3389/fmicb.2023.1060891

**Published:** 2023-03-07

**Authors:** Simone Marini, Christina Boucher, Noelle Noyes, Mattia Prosperi

**Affiliations:** ^1^Department of Epidemiology, University of Florida, Gainesville, FL, United States; ^2^Department of Pathology, University of Florida, Gainesville, FL, United States; ^3^Department of Computer and Information Science and Engineering, University of Florida, Gainesville, FL, United States; ^4^Department of Veterinary Population Medicine, University of Minnesota, St. Paul, MN, United States

**Keywords:** antibiotic resistance, gene variants, metagenomics, high-throughput sequencing, bioinformatics, statistical learning

## Abstract

Characterization of antibiotic resistance genes (ARGs) from high-throughput sequencing data of metagenomics and cultured bacterial samples is a challenging task, with the need to account for both computational (e.g., string algorithms) and biological (e.g., gene transfers, rearrangements) aspects. Curated ARG databases exist together with assorted ARG classification approaches (e.g., database alignment, machine learning). Besides ARGs that naturally occur in bacterial strains or are acquired through mobile elements, there are chromosomal genes that can render a bacterium resistant to antibiotics through point mutations, i.e., ARG variants (ARGVs). While ARG repositories also collect ARGVs, there are only a few tools that are able to identify ARGVs from metagenomics and high throughput sequencing data, with a number of limitations (e.g., pre-assembly, *a posteriori* verification of mutations, or specification of species). In this work we present the *k*-mer, i.e., strings of fixed length *k*, ARGV analyzer – KARGVA – an open-source, multi-platform tool that provides: (i) an *ad hoc*, large ARGV database derived from multiple sources; (ii) input capability for various types of high-throughput sequencing data; (iii) a three-way, hash-based, *k*-mer search setup to process data efficiently, linking *k*-mers to ARGVs, *k*-mers to point mutations, and ARGVs to *k*-mers, respectively; (iv) a statistical filter on sequence classification to reduce type I and II errors. On semi-synthetic data, KARGVA provides very high accuracy even in presence of high sequencing errors or mutations (99.2 and 86.6% accuracy within 1 and 5% base change rates, respectively), and genome rearrangements (98.2% accuracy), with robust performance on *ad hoc* false positive sets. On data from the worldwide MetaSUB consortium, comprising 3,700+ metagenomics experiments, KARGVA identifies more ARGVs than Resistance Gene Identifier (4.8x) and PointFinder (6.8x), yet all predictions are below the expected false positive estimates. The prevalence of ARGVs is correlated to ARGs but ecological characteristics do not explain well ARGV variance. KARGVA is publicly available at https://github.com/DataIntellSystLab/KARGVA under MIT license.

## 1. Introduction

Bacterial antimicrobial resistance is a global threat to human health and to numerous ecosystems, responsible for over 1 million and associated to over 4 million of peoples’ deaths annually (2019 estimate) worldwide (Murray et al., 2022), disruption of livestock production, and environmental contamination (Iwu et al., 2020). Resistance in bacteria can manifest naturally, evolve through genetic mutations, or be acquired through gene transfer. While antibiotic susceptibility testing through *in vitro* cultures is the standard in microbiology research and in clinical/veterinary care settings, high-throughput – targeted and metagenomics – sequencing is becoming a promising alternative, at a relatively low cost and fast turnaround time (Gu et al., 2019; de Abreu et al., 2021). The characterization of antibiotic resistance genes (ARGs) from metagenomics as well as cultured bacterial samples through high-throughput sequencing involves development of computational approaches to process large experimental data, up to the terabyte scale, as well as biological annotation of existing and new ARGs into curated database resources (Boolchandani et al., 2019).

Several public ARG databases are actively maintained, e.g., the comprehensive antibiotic resistance database (CARD; Alcock et al., 2020), MEGARes (Doster et al., 2020), the national database of antibiotic resistant organisms (NDARO; Sayer et al., 2020), ResFinder (Bortolaia et al., 2020), and the structured antibiotic resistance gene (SARG) database (Yin et al., 2018), providing access to thousands of gene entries and functional annotations. There is substantial overlap among the databases, however, and it is nontrivial to compare the annotations due to differences in ontology used. For instance, MEGARes uses a tree-based hierarchical structure to represent antimicrobial chemical classes, biological mechanisms, and operon-level gene groups; conversely, CARD uses a reticulate structure. Numerous tools for ARG identification from high-throughput sequencing data are available that rely on one or more of these databases, including methods that are based on: sequence alignment, e.g., AMRPlusPlus (Doster et al., 2020); *k*-mers (strings of fixed length *k*), e.g., ResFinder (Bortolaia et al., 2020), KARGA (Prosperi and Marini, 2021), and AMR-meta (Marini et al., 2022); hidden Markov models, e.g., Meta-MARC (Lakin et al., 2019), Resfams (Gibson et al., 2015); and other machine learning algorithms, e.g., DeepARG (Arango-Argoty et al., 2018).

One crucial problem in ARG identification that has gone largely unexplored for metagenomic data is the detection of *ARG variants* (ARGVs; Coculescu, 2009; Sultan et al., 2018; Prosperi et al., 2019). ARGVs are constituted by point mutations that allow regular chromosomal genes (e.g., housekeeping genes, topoisomerases) to confer resistance to one or more antimicrobials. It is particularly challenging to detect this type of resistance because both the gene and the mutation(s) must occur. Thus, only a few methods are capable of flagging ARGVs and/or verifying the presence of point mutations. AMRPlusPlus 2.0 flags genes that require variant confirmation, but leaves this additional analysis to the user. Other algorithms might require partial pre-assembly and protein translation, as was recommended in CARD’s resistance gene identifier (RGI) prior to version 6.0.0 to avoid over-reporting alignment to susceptible alleles; or they might be limited to a number of bacterial strains that have to be specified at runtime, such as PointFinder. This is a critical issue as ARGVs can encompass a substantial part of ARG databases. For example, 6% of the ARGs in MEGARes are ARGVs.

Here, we present the *k*-mer antibiotic resistance gene variant analyzer (KARGVA). KARGVA is a multi-platform, open-source software specifically designed to confirm the presence of point resistance mutations in chromosomal genes from either metagenomics or whole genome sequencing data. KARGVA is built after – and it is intended to be used in conjunction with – our prior approach for regular ARGs, which is KARGA (Prosperi and Marini, 2021). KARGVA merges different ARGV databases and utilizes an efficient three-way, hash-based approach to mutually link point mutations, *k*-mers, and genes. It is equipped with a statistical approach to filter false positives and rank multiple, plausible ARGV candidates. Besides the algorithmic innovation, KARGVA provides advantage over existing tools since it relaxes the need to perform assembly and/or identify species before analysis, and automatically confirms the presence of required mutations during the analysis.

On semi-synthetic data, KARGVA provides high accuracy with sequencing errors or gene mutations and demonstrates robustness with respect to false positive sets. On a large collection of metagenomic data collated by the MetaSUB consortium, KARGVA identifies more ARGVs than AMRPlusPlus, RGI, and PointFinder, and its predictions exhibit a low false positive rate (estimated on a semi-synthetic benchmark set).

## 2. Methods

**Figure 1:**
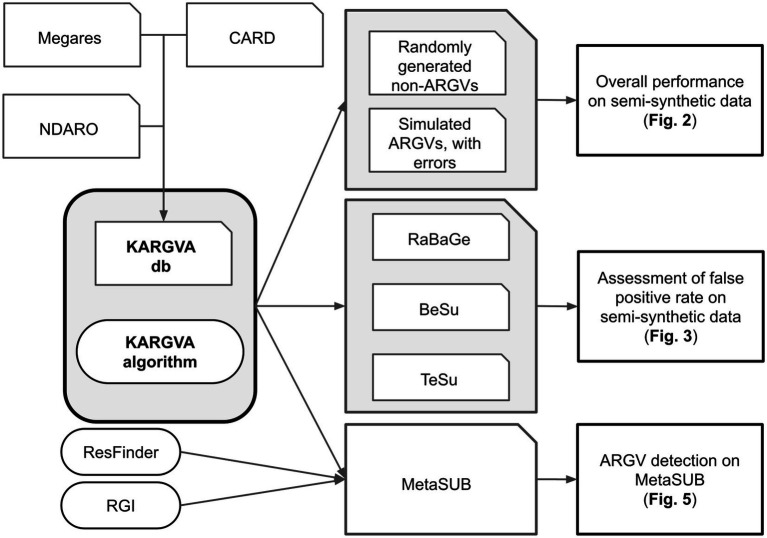
Development of KARGVA: The KARGVA database is collated from different sources. Benchmarks on semi-synthetic data: a first semi-synthetic dataset is used to benchmark KARGVA against randomly generated non-ARGVs and simulated ARGVs with errors; and three other semi-synthetic datasets are used to benchmark KARGVA against false positives. Evaluation on real metagenomics data: KAGVA is applied to real metagenomics data and the results are compared with existing ARGV prediction algorithms.

We proceed as follows: First, we collate and manually curate ARGVs from different sources into the KARGVA database and develop the KARGVA algorithm; second, we benchmark KARGVA on semi-synthetic datasets with a known ground truth; finally, we validate KARGVA on real metagenomics data. This process is depicted in Figure 1.

### 2.1. Database collation

KARGVA integrates ARGV sequences from three different ARG databases: CARD,^1^ MEGARes,^2^ and NDARO.^3^ We collect all the protein entries for NDARO. For CARD, we extract ARGVs from the protein variant model, including single resistance variants, multiple resistance variants (with the exclusion of duplications), nonsense mutations, and high confidence *M. tuberculosis* ARGVs (integrated in CARD from the ReSeqTB database).^4^ MEGARes ARGVs are compiled through external header and sequence mapping to one of the other sources with available point mutation information. The merged database is made partially non-redundant by collapsing sequences that are identical at the amino acid level. However, multiple mutations are unmodified from the original sources. Thus, if a gene can become resistant through multiple independent point mutations or different combinations of mutations, e.g., the wild-type gene MKRIK mutates into MKRVK or MKRPK to become resistant, all resulting ARGVs appear separately in the database. This is done on purpose to respect the natural occurrence of amino acid variations and their combinations as observed in real-world samples.

Sequence headers and antibiotic resistance ontology metadata, e.g., MEGARes’ class/mechanism/group hierarchy or NDARO’s antibiotic compounds, are pooled together. Since the ontologies of MEGARes, and CARD differ, and NDARO does not use a standardized annotation, KARGVA can report ARGVs with one ontology/annotation term, or more than one if the same ARGV comes from multiple databases.

### 2.2. Classification approach and statistical scoring

The classification of a DNA sequence read as belonging to an ARGV is carried out at the amino acid level, comparing the *k*-mer spectrum of a given protein-translated query sequence with the *k*-mer spectrum of the ARGV database and, separately, of all *k*-mers that include point mutations associated with antibiotic resistance. Since raw high-throughput data is at the nucleotide level, each sequence read is paired to its reverse complement, translated into amino acids using all six reading frames, and then queried against the merged ARGV database. The reason for performing reverse complements and protein translation in all reading frames is due to the fact that mutations of the AMR genes are annotated at the amino-acidic level, and thus we need to identify the right coding frame for a given read. Of note, since reads can contain errors (including frameshifts), we do not base the choice on stop codons, but we pick the translation with highest number of *k*-mer matches and mutation matches at the amino-acidic level, using the algorithm below.

For each query, first it is verified to determine whether its *k*-mer spectrum contains resistance mutations, by comparing it with each ARGV’s subset of *k*-mers that include at least one resistance mutation, out of the complete *k*-mer spectrum of each ARGV. All ARGVs for which at least one positive match of point mutations is found are retained as candidates. Second, the most probable ARGVs are chosen on the basis of the highest relative prevalence of point mutation *k*-mers as well as all *k*-mers that are included in the whole *k*-mer spectrum of an ARGV, but do not necessarily contain point mutations. Since *k*-mers might not be unique to ARGVs, the prevalence scores are weighted by the *k*-mer multiplicity in different genes, e.g., if a *k*-mer is present in three different ARGV genes, its weight is one third as compared to a *k*-mer that is found in one ARGV gene only. A combined score is the result of the probabilistic sum (P_combined_ = P_1_ + P_2_ – P_1_×P_2_) of the two matching measures (i.e., P_1_ represents the fraction of *k*-mers of the read that match a gene and P_2_ represents the proportion of the subset of matching *k*-mers that contain resistance mutations) and decrees the final ARGV ranking.

We also use a statistical test that checks how many *k*-mer matches relative to point mutations could be due to chance given a specific sequence length and *k* value. In this way, we are able to filter out false positive classifications at a desired level of confidence. Our procedure calculates the empirical distribution of point mutation *k*-mer occurrences for a sufficiently number of random queries, and draws the probability threshold on the basis of the percentile distribution counts, resembling an exact formula previously introduced theoretically (Prosperi et al., 2012), and then later implemented. In brief, the test simulates a number of random reads and compares their *k*-mer spectrum against the AMR database, deriving a count distribution of *k*-mer matches. A given percentile of this distribution, e.g., 95th or 99th, is used as a threshold to flag false positives. For KARGVA, the *p*-value for false positive rejection is set to 0.01, and the number of query randomizations is set to 12,500 (although it can be changed by the user). This parameter has been empirically optimized over 200,000 tests to accommodate a broad range of read lengths and *k*-mer lengths (from 3 to 41 nucleotides). We showed that the probability density function is degenerate until *k* = 11, which is the minimum *k* we allow, and then the 99th percentile quickly approaches the unity from *k* = 23 and higher (Prosperi and Marini, 2021). As the ARGV database is only in part non-redundant, there can be cases when more than one ARGV exhibits an optimal score not by chance. To account for these situations, KARGVA reports all scores that are within 5% of the optimal one. Thus, for each sequence read, either no classification to ARGV or classification to one or more ARGVs is provided, with the statistical summaries.

Concomitantly to the per-read classification, KARGVA updates a whole-sample classification, as follows: each time a protein sequence query is assigned to one or more ARGVs, its *k*-mer spectrum is used to update the overall *k*-mer content match for each ARGV in the database. There can be also cases when the *k*-mer content of a read significantly overlaps with that of a region of one or more ARGVs where no required mutations are present, and the read is still used to update the coverage and depth of said ARGV. However, only ARGVs whose resistance mutations are confirmed and pass the statistical filtering are eligible for output, regardless the overall *k*-mer matches. Thus, after processing all reads, KARGVA outputs sample-level coverage and depth summaries for all ARGVs in the database. The coverage is quantified as the fraction of *k*-mers over the total number of distinct *k*-mers in the gene (weighting multiplicity), whilst the depth is the average number of times a *k*-mer is covered.

### 2.3. Data structures and implementation

Three main relational structures are created by indexing: (1) ARGV identifiers linked to *k*-mers (one-to-many); *k*-mers containing point mutations linked to ARGV identifiers (one-to-many); (2) non-mutant *k*-mers linked to ARGV identifiers (one-to-many); and (3) ARGV identifiers linked to all their *k*-mers along with frequencies (many-to-one-to-many). The *k*-mers are placed in a hash table; therefore, there is a unique hash code identifying a unique *k*-mer. However, a unique *k*-mer can be found in multiple genes with a certain multiplicity. The multiple genes are stored in array lists whose pointers are the values of the said *k*-mer hash table. The *k*-mer multiplicity is stored in secondary hash tables for each AMR gene. The code is implemented in Java,^5^ using legacy data structures (HashMaps and ArrayLists) for the aforementioned relations. The number of *k*-mers of the merged databases has been calculated in advance to verify that there would not be problems with hash collisions or memory usage, even by doubling the database size, without the need to resort to more efficient data structures. KARGVA accepts as input both uncompressed and compressed FASTQ files (recognized automatically through file extension, or as indicated by the user in the command line). The source code is hosted in GitHub^6^ and publicly available at: https://github.com/DataIntellSystLab/KARGVA under MIT license. The GitHub folder includes a detailed README with information about database version, set up of input parameters (*k*-mer length, number of random queries to determine false positive threshold, ARGV reference file, read file, single vs. multiple ARGV scoring per read), and output file description.

### 2.4. Experimental setup

Four semi-synthetic datasets are made by simulating high-throughput metagenomic sequencing experiments in different settings, and are used for parameter optimization, performance evaluation and robustness assessment. Specifically, KARGVA is optimized on different values of *k* (between 21 and 45 nucleotides), to determine the best tradeoff between the false positive rate and the false negative rate, with respect to non-ARGV sequences, and ARGV sequences that may carry non-ARGV mutations, gene rearrangements, or sequencing errors.

#### 2.4.1. First simulated dataset

The first simulated dataset consists of FASTQ files made of: (i) reads drawn from ARGV databases with non-ARGV mutations or sequencing errors up to a 15% rate; (ii) reads from ARGV databases with a two-point transposition (with/without reversion), each 50% of the read length; and (iii) non-ARG reads, generated uniformly at random.

#### 2.4.2. Second simulated dataset (RaBaGe)

The second read set (semi-synthetic) is generated from 5,000 randomly picked RefSeq (O’Leary et al., 2016) bacterial genes (RaBaGe) that did not match any sequence in MEGARes with a BLAST search (*e*-value = 10), putatively susceptible to all antibiotics.

#### 2.4.3. Third and fourth simulated datasets (BeSu and TeSu)

The third semi-synthetic dataset is a specific betalactam-susceptible (BeSu) dataset obtained from PATRIC (Pathosystems Resource Integration Center) web repository (Davis et al., 2020), where an antibiotic susceptibility test is available, made by clipping genes of bacterial genomes that (1) are among the top 10% in terms of the numbers of different betalactam antibiotics they were resistant against; and (2) exhibit medium-high similarity to MEGARes genes (BLAST *e*-value ≤ 0.01, percent identity between 70 and 90%). The fourth semi-synthetic dataset is a specific tetracycline-susceptible (TeSu) dataset, collated in the same way as BeSu.

In summary, the first dataset is used to assess the classification performance on different error rates as well as gene rearrangements; while RaBaGe, BeSu, and TeSu are all “negative” datasets according to the antibiotic susceptibility testing or (non)match with MEGARes. An ARGV classifier should therefore *not* find antibiotic resistance genes in these datasets, unless the genes were present but not expressed. RaBaGe covers the spectrum of all genes, while BeSu focus on genes that are very similar to ARGVs but present different mutations. With all datasets we assess and optimize the false positive rate of KARGVA by varying *k* and gene coverage threshold. The semi-synthetic datasets are simulated using InSilicoSeq software (v 1.4.4) with presets for Illumina (read length 151; Gourlé et al., 2019).

We finally evaluate KARGVA on real experimental data, using metagenomic experiments from samples collected by the MetaSUB consortium (Mason et al., 2016). MetaSUB is an international project that collects surface samples from public transportation systems, and collates metagenomic sequencing of urban microbiomes with the goal *“to build a molecular profile of cities around the globe to improve their design, functionality, and impact on health.”* We replicate in part the work presented by Danko et al. (2021), who applied AMRPlusPlus to detect both ARGs and ARGVs, and provided an abundance, density and correlation analysis among sampled cities’ metagenomes in relation to their distance and distribution of resistance genes. To extract the species present in each sample, we utilize Kraken2 (Wood et al., 2019) and its standard database version as of September 04, 2019. In addition to correlation and density maps for sample characteristics by ARGs and ARGVs, we fit a random forest (RF) to evaluate how much variance in ARGVs is explained by the sample attributes –the city of origin, surface material (manually curated, grouping materials that are not present in at least five cities and in a total of 25 samples into an “other” category), type of sample (air, environmental, and unknown), number of total reads per sample, and bacterial species, classified with Kraken2. We use R^7^ with packages caret and ranger, optimizing the number of trees and number of splits at each tree node *via* grid search. RF variable importance is evaluated *via* permutation. Prediction performance is assessed through 10-fold cross validation, measuring the root mean squared error (RMSE) and mean absolute error (MAE).

We further compare KARGVA with AMRPlusPlus 2.0, RGI 5.1.1 and PointFinder 4.2. AMRPlusPlus is an alignment-based method that identifies ARGs as well as candidate ARGVs (that require confirmation of mutations) from high-throughput read data using MEGARes. RGI is also alignment-based, but it uses protein-translated queries and CARD. PointFinder is a hybrid method that uses alignment and *k*-mers. In order to evaluate RGI and PointFinder, the raw read files are assembled using metaSPAdes 3.15.3 (recommended parameters), after quality control, filtering and adapter trimming preprocessing (Nurk et al., 2017). Furthermore, as PointFinder requires to specify an input species, we run it for all the supported species, merging its output and removing duplicates. For comparing ARGV predictions in this work – since ARGV classification tools may use different ontology – we manually review all the antibiotic resistance ontology/prediction annotations of KARGVA, RGI, and PointFinder, using MEGARes classes as a common reference. All terminology for which we are able to find a correspondence is retained (see Supplementary material).

All tests are run on University of Florida’s HiPerGator computing cluster^8^ on nodes with 4 Intel Xeon CPUs at 2.00 Ghz with 16 GB RAM.

## 3. Results

### 3.1. Characteristics of the merged database

The KARGVA ARGV database integrates 1,159 single-point, and 95 multiple-point ARGVs from CARD (January 2021 release); and 654 single-point ARGVs from NDARO 3.10, respectively. In MEGARes, ARGVs are not provided with information on the variant location, but we are able to align 1,173 MEGARes’ ARGVs with CARD, and 147 with NDARO. After merging identical sequences, the final KARGVA database includes 1,781 ARGVs. Out of the total, 95.5% of sequences contain just one point mutation conferring antibiotic resistance, 3.3% contain two, 1.1% contain three or four, and 2.5% contain stop codons. KARGVA reports ARGV annotations in accordance with all original database ontologies; in the case of a sequence that is present in more than one database, all individual annotations are provided together.

### 3.2. Benchmarks on semi-synthetic data

**Figure 2:**
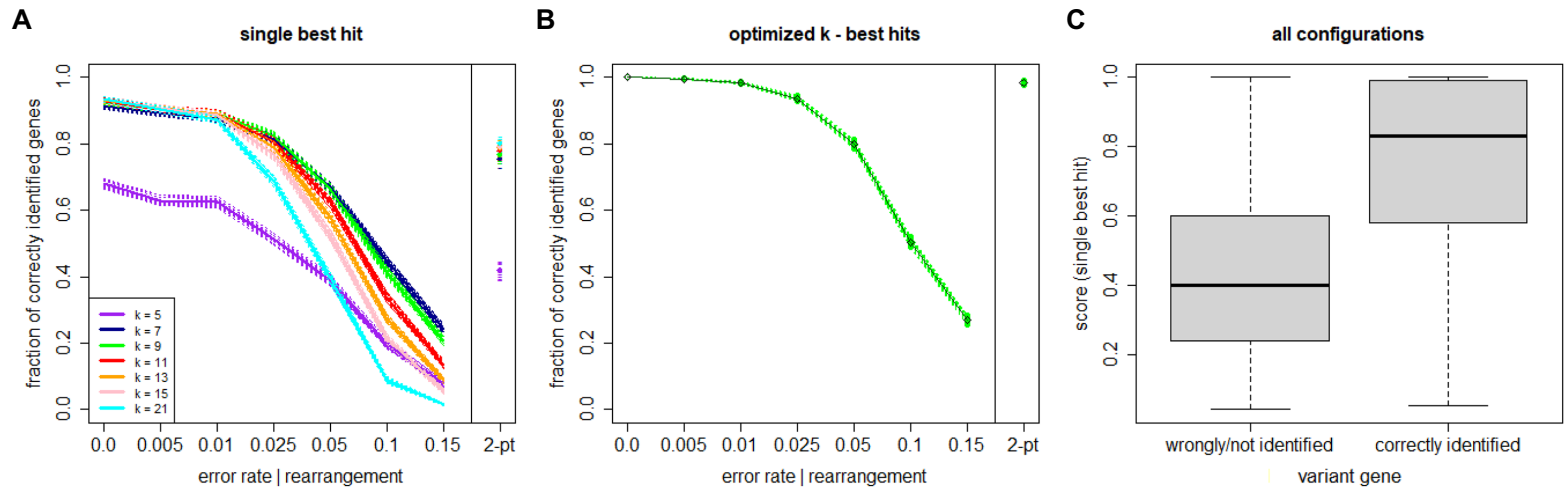
KARGVA performance on semi-synthetic data (varying error/nucleotide change rates and gene rearrangements) in identifying genes of bacteria conferring antibiotic resistance through point mutations. **(A)** shows performance for a single best match, stratified by parameter *k* value; **(B)** shows performance after *k* optimization, using all optimal best scoring matches; **(C)** shows box-and-whisker plot distribution of algorithm’s scores on all test configurations, stratified in accordance with ground truth. Shaded areas and whiskers represent 95% confidence intervals. Numbers used for this figure are provided in the Supplementary material.

The first semi-synthetic dataset is used to optimize the *k* value and to assess the single best match performance across different sequencing error rates (or more in general as any nucleotide change from the original ARGV sequence, including an actual mutation from a different bacterial strain). Each simulation comprises 25,000 reads of length of 151 bases. Figure 2A displays the accuracy curves (with 95% confidence intervals drawn across simulations) stratified by *k* value. Numbers are reported in the Supplementary material. Larger *k* values are more accurate at lower error rates, and *vice-versa*. The best tradeoff is given by *k* = 9 amino acids (i.e., 27 nucleotides), yielding accuracies well over 80% for error or base change rates up to 2.5%. Figure 2C illustrates the distribution of scores for the single best match across all experimental configurations, stratified by the classification correctness. The distributions clearly indicate that the proposed score has very high discriminative ability, yielding a median (IQR) value of 0.83 (0.59–0.99) for the correctly identified genes vs. 0.41 (0.21–0.60) for the wrongly/non-identified entries. As explained, more than one ARGV can have optimal score, so we can evaluate all best scoring ARGVs. Figure 2B shows the performance of our method using the best *k* and the top scoring matches. The accuracy is 99.2% for error rates up to 1, 93.5% at 2.5, 79.8% at 5, 50.3% at 10, and 26.9% at 15%. For 2-point gene rearrangements, the overall accuracy is 98.2%. Note that the accuracy statistics are calculated on ARGV (either with a given error rate or 2-point rearrangement) upon the false positive testing, which filters out the non-ARGV reads randomly generated. Our method correctly identified 100% of random reads. Thus, the accuracy is indeed a sensitivity with ideal 100% specificity.

**Figure 3:**
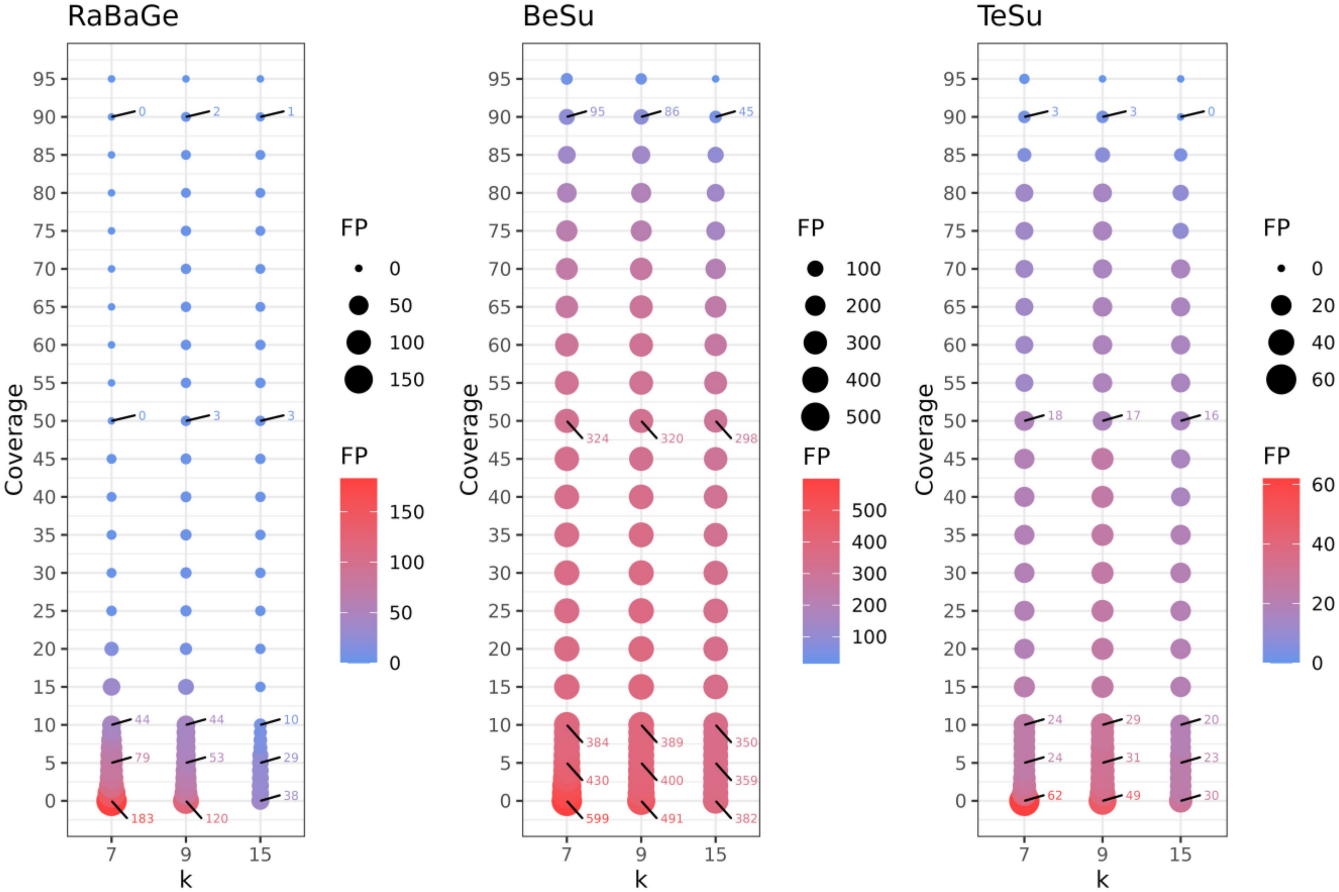
Assessment of KARGVA’s false positive rate on semi-synthetic data by varying *k*-mer length and gene coverage threshold. The *X*-axis represents the *k* value (in amino acids, e.g., *k* = 9 means 27 nucleotides), the *Y*-axis represents the gene coverage portion (%), and the bubble size represent the false positive counts. RaBaGe, BeSu, and TeSu are synthetic datasets assembled by random bacterial genes (RaBaGe), and PATRIC genome fragments exhibit ingmedium-high similarity to MEGARes betalactamase (BeSu) or tetracycline (TeSu) genes.

The other three semi-synthetic datasets help us determine the robustness of KARGVA with respect to false positive rate on more realistic bacterial metagenomics data. For RaBaGe, we select over 5,000 genes with length >500 nucleotides that meet the antibiotic susceptibility criteria, and generate 500,000 reads, for BeSu we obtain 4.2 million reads, while for TeSu 355,170 reads. We benchmark KARGVA over multiple parameter combinations, varying the minimal required gene fraction coverage between 0.00 and 0.95, and considering *k* equal to 7, 9, 15 (i.e., 21, 27, 45 nucleotides). For each parameter configuration and dataset, we calculate the false positive score (FPS), measured as # of detected ARGVs/# of reads. As shown in Figure 3, even at low coverage thresholds and small *k* values, the FPS is low. With any coverage fraction and *k* = 7, the FPS is 3.7 × 10^−4^ in RaBaGe, 1.5 × 10^−4^ in BeSu, and 1.7 × 10^−4^ in TeSu. With the most conservative coverage of 0.95 and *k* = 15 (i.e., long conserved stretches), FPSs is 2 × 10^−6^, 3.8 × 10^−6^, and 1.1 × 10^−5^ for RaBaGe, BeSu, and TeSu, respectively. We then set the KARGVA to default configuration with coverage of 0.8 and *k* = 9, which yields FPS rates all below 5 in 100,000, specifically 4 × 10^−6^, 4.5 × 10^−5^, and 4.2 × 10^−5^ for RaBaGe, BeSu, and TeSu. We expect real metagenomics data to be in-between the “easy” (RaBaGe) and “hard” (BeSu, TeSu) negative datasets, and therefore we estimate the expected KARGVA FPS on metagenomics data to be in the [10^−5^, 10^−6^] range when used with default settings. We also test RGI and PointFinder on these three negative datasets. RGI shows a worse FPS, yielding at least twice false positives than KARGVA. Over RaBaGe, BeSu, and TeSu, RGI FPSs are 6 × 10^−6^, 9 × 10^−5^, and 1.2 × 10^−4^, while PointFinder shows the best FPSs with 4.8 × 10^−7^, 3.3 × 10^−6^, and less than 2.8 × 10^−6^, i.e., no findings over TeSu. Note that both RGI and PointFinder are run on contigs assembled by metaSPAdes, and not on the original read sets.

### 3.3. Evaluation on real metagenomics data

**Figure 4:**
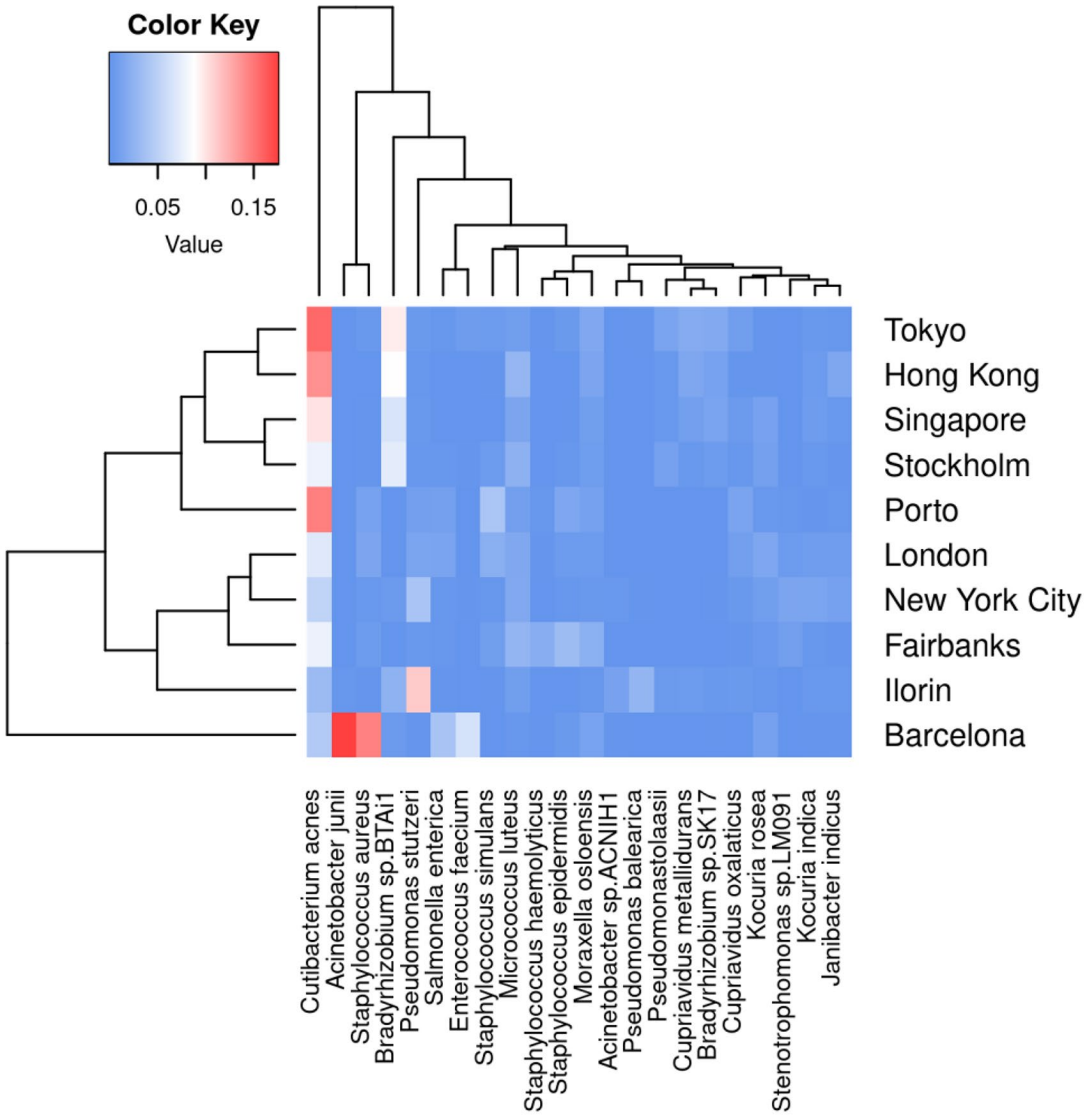
Average per-sample frequency of bacterial reads, considering the top 10 cities per number of sample, and the top-5 species per city. The color key represents the prevalence in [0, 1] scale of each species.

Next, we run KARGVA, AMRPlusPlus, metaSPAdes+RGI and metaSPAdes+PointFinder on the metagenomics global data (surface samples from public transportation) from the MetaSUB project. The MetaSUB FASTQ files available for public download are filtered for human DNA. Of 4,305 paired short read files, 3,758 come with a matched metadata record and belong to a city with at least 25 samples, and can thus be processed (i.e., provide an output) by all the algorithms we used. Table 1 shows sample/isolate characteristics for the MetaSUB files selected and analyzed, considering the top-10 cities in terms of total number of samples, with summaries of the top-5 most frequent species as classified by Kraken2, and the mean, median (interquartile) number of ARGs detected by AMRPlusPlus and of ARGVs detected by KARGVA. Figure 4 shows the relationships between species abundance and city, considering the top-5 species. Out of 5,053 unique species detected, 22 make the top-5. *Cutibacterium acnes* is the most abundant in terms of average per-sample reads in eight of the top-10 cities. Of note, a considerable fraction of the reads (28–55%) cannot be assigned to a species using the standard Kraken2 database.

**Table 1:** Summary of MetaSUB study data stratified by city (top-10 by number of isolates, others aggregated).

City	No. of samples	Top-5 species	Mean; Median (IQR) no. of ARGs per sample	Mean; Median (IQR) no. of ARGVs per sample	Median (IQR) % of unaligned reads per sample
Hong Kong	770	*Cutibacterium acnes; Bradyrhizobium* sp. *BTAi1; Micrococcus luteus; Cupriavidus metallidurans; Janibacter indicus*	21.28; 17 (9, 28)	3.9; 2 (2, 3)	33.02 (26.59,42.87)
London	635	*Cutibacterium acnes; Staphylococcus simulans; Micrococcus luteus; Kocuria rosea;* *Staphylococcus aureus*	18.57; 4 (1, 14)	23.85; 2 (2, 4)	42.18 (32.85, 48.41)
New York City	493	*Cutibacterium acnes; Pseudomonas stutzeri; Micrococcus luteus; Stenotrophmonas* sp. *LM091; Kocuria indica*	36.92; 20 (9, 41)	25.01; 4 (2, 23)	28.14 (18.42, 43.07)
Ilorin	264	*Pseudomonas stutzeri; Cutibacterium acnes; Pseudomonas balearica; Bradyrhizobium* sp. *BTAi1; Acinetobacter* sp. *ACNIH1*	57.9; 31.5 (9.75, 75.75)	36.5; 13 (2, 56.5)	43.38 (25.79, 55.16)
Singapore	179	*Cutibacterium acnes; Bradyrhizobium* sp. *BTAi1; Micrococcus luteus; Bradyrhizobium* sp. *SK17;* *Kocuria rosea*	18.58; 9 (4, 17.5)	12.75; 2 (2, 4)	43.19 (32.68, 54.12)
Tokyo	148	*Cutibacterium acnes; Bradyrhizobium* sp. *BTAi1; Cupriavidus metallidurans;; Bradyrhizobium* sp. *SK17; Moraxella osloensis;*	40.29; 13.5 (6, 32.5)	26.23; 2 (2, 8)	32.53 (22.33, 55.41)
Barcelona	116	*Acinetobacter junii; Staphylococcus aureus: Enterococcus faecium; Cutibacterium acnes; Salmonella enterica*	71.53; 10 (2.75, 24.5)	91.7; 4 (2, 15)	36.49 (18.89, 59.49)
Stockholm	110	*Cutibacterium acnes; Bradyrhizobium* sp. *BTAi1; Micrococcus luteus; Pseudomonas stolaasi;* *Kocuria rosea*	5.18; 2 (0, 6)	2.07; 2 (2, 2)	54.55 (48.58, 58.99)
Porto	107	*Cutibacterium acnes; Staphylococcus simulans; Staphylococcus epidermis; Cupriavidus oxalaticus;* *Staphylococcus aureus*	5.8; 2 (0, 6)	23.52; 2 (2, 2)	53.11 (45.77, 61.76)
Fairbanks	95	*Cutibacterium acnes; Staphylococcus epidermidis; Micrococcus luteus; Moraxella Osloensis; Staphylococcus haemoliticus*	26.44; 13 (4, 35.5)	7.59; 2.5 (2, 15)	43.55 (25.61, 55.05)
Other (aggregated)	841	*Pseudomonas stutzeri; Cutibacter iumacnes; Bradyrhizobium* sp. *BTAi1; Acinetobacter woffii; Moraxella osloensis*	32.58; 11 (4, 25)	20.02; 2 (2, 5)	45.44 (32.16, 53.57)

We report the five most frequent bacterial species from each location (based on per sample prevalence), along with the median percentage of unclassified samples, as found by Kraken 2.0. Antibiotic resistance genes (ARGs) are identified using AMRPlusPlus 2.0, while ARGVs are identified using KARGVA. IQR, interquartile range.

**Figure 5:**
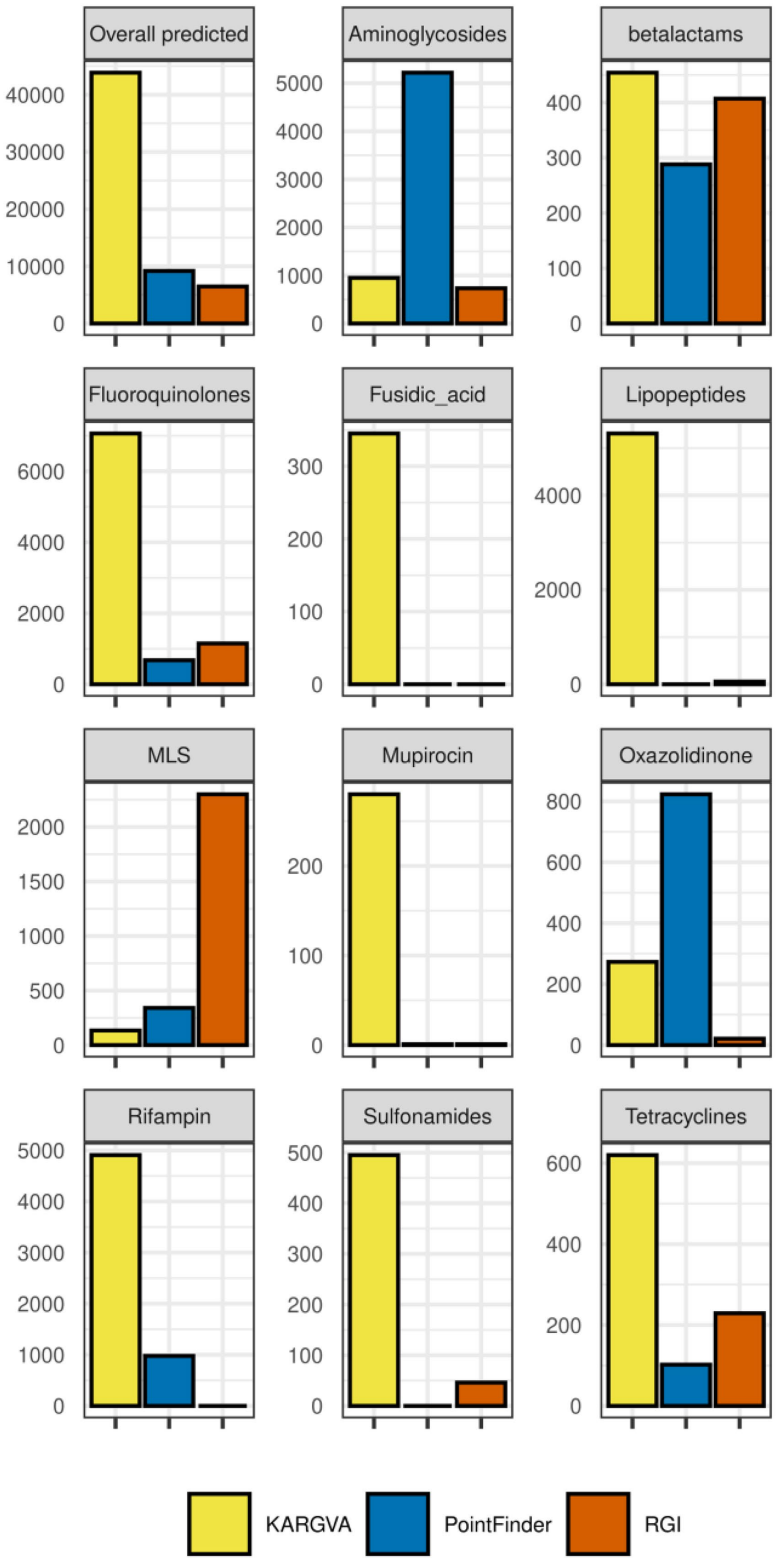
ARGVs detected in the MetaSUB metagenomics datasets (*n* = 3,758 with available metadata) by KARGVA, RGI, PointFinder, both overall and per class.

In order to assess the reliability of ARGV findings, we compare in detail the ARGV detection by KARGVA with respect to that of AMRPlusPlus, RGI, and PointFinder. In Figure 5, we calculate ARGV counts for all algorithms overall (i.e., total number of ARGVs, independently from the class) and per-class. Through the ontology/annotation mapping described in the methods, we identify 11 MEGARes classes that can be predicted by all three algorithms: amingoglycosides; betalactam; fluoroquinolones; fusidic acid; lipopeptides; macrolide, lincosamide and streptogramin (MLS); mupirocin; oxazolidnone; rifampin; sulfonamides; and tetracyclines. For this reason, the per-class comparison must be limited to the classes all algorithms can predict. Of note, while by design KARGVA has a single MEGARes class assigned to each prediction, RGI and PointFinder might have multiple, i.e., multiple output terms can match the same MEGARes class in a sample. We therefore allow RGI and PointFinder to count multiple times if the annotation terms of their predictions match with more than one class (see Supplementary material). Overall, KARGVA finds 43,846 ARGVs, ~4.8 times more than PointFinder (9,185) and ~6.8 more than RGI (6,472). KARGVA retrieves the highest number of ARGVs in 8 out of 11 considered classes, the exceptions being aminoglycosides (highest: PointFinder), MLS (highest: RGI), and Oxazolidinone (highest: PointFinder). Although it is not possible to directly transpose the FPS from the synthetic datasets to the MetaSUB results, we expect RGI to find more false positives then the other two algorithms, and PointFinder to be the most conservative. For a reference, AMRPlusPlus yields over 100,000 ARGVs that need SNP confirmation; KARGVA, RGI, and PointFinder are all well below this value.

We then analyze how KARGVA’s ARGVs relate to the ecological characteristics of the samples and the ARG distributions. Using the top-10 city set, the per-sample median and mean number of retrieved ARGs by AMRPlusPlus (mean range: 2–31.5; median range: 5.18–71.53) are comparable to the retrieved ARGVs by KARGVA (mean range: 2–13; median range: 3.9–91.7), and they are strongly correlated (Spearman’s correlation for mean and median, respectively: 0.63; 0.89). Note that we cannot achieve a perfect replication of the original ARG analysis presented by Danko et al. (2021), since the original MetaSUB analysis used MEGARes 1.0.1 and Bowtie 2.3.0. Instead, we apply filtering criteria on the cities based on sample size, and use the most up-to-date AMRPlusPlus 2.0 pipeline, which employs MEGARes 2.0 and BWA, along with specific preprocessing (Trimmomatic) and post-processing (Bedtools, SNPfinder), finalizing with the ResistomeAnalyzer. Nonetheless, there is consistency in the overall output, with more positive identifications expected, since MEGARes 2.0 contains more genes than the prior release.

**Figure 6:**
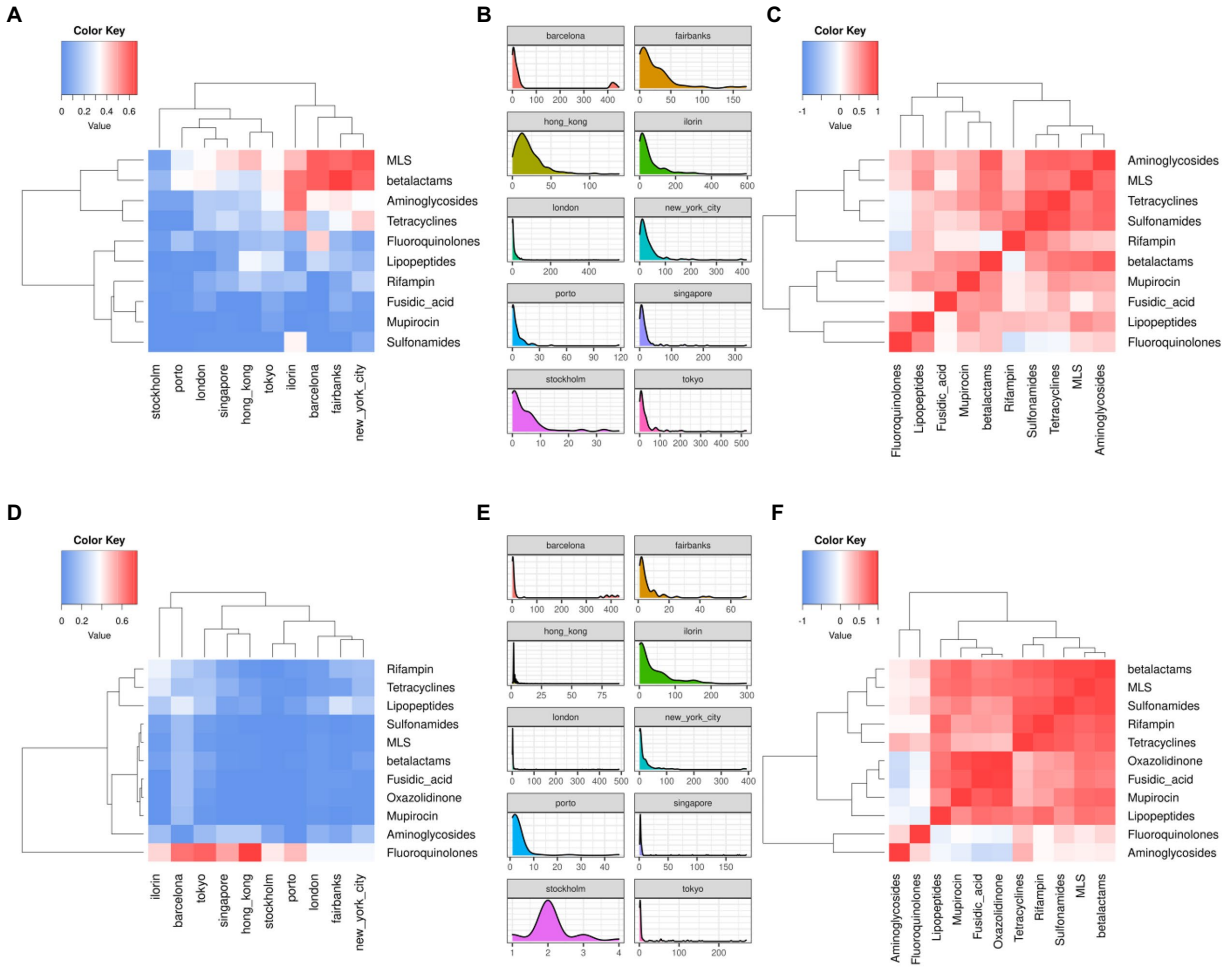
Correlation heatmaps (fraction of counts per antibiotic resistance class over the city total) and density plots of antibiotic resistance genes (ARGs, Panels **A–C**) and ARG variants (ARGVs, Panels **D–F**), among the top-10 cities and resistance types using the MetaSUB annotated samples (*n* = 2,917).

By stratifying the distribution of ARG and ARGV findings per city and antibiotic class, we check if there are relevant correlations between city and class or between classes. Figure 6 shows: the per-city ARG and ARGV (panels B and E) distributions, the per-city ARG and ARGV (panels A and D) class profiles – defined as the fraction of the per-class counts over the total counts of a city – and the per-class correlation (Spearman) based on the 10 city profiles. Large fractions of ARG counts per-city come from MLS, betalactam, and aminoglycoside classes, while the highest fractions of ARGV counts come from fluoroquinolones. The class-to-class correlation structures are different between ARGs and ARGVs. For instance, fluoroquinolones and lipopeptides, as well as MLS and aminoglycosides ARGs are found often together, while the correlation is low in ARGVs, where fluoroquinolones and aminoglycosides tend to cluster apart from the others. Of note, there are differences among the ARG/ARGV classes reported. Some resistance classes, such as Oxazolidinone, are not present as ARGs, since MEGARes and KARGVA annotations have only a partial overlap.

After correlation and density analysis, we fit the RF model to predict the number of ARGVs per samples given the city of origin (24 cities), surface material, type of sample, total reads, and bacterial species (5,054 species by Kraken2). Using 250 trees and varying the number of attributes considered at each split, performance varies from 34.6 ± 1.41 (RMSE) and 14.2 ± 1.41 (MAE) with 2 splits per node, to 12 ± 2.3 (RMSE) to 4.3 ± 0.38 (MAE) with 5,089 splits per node, i.e., considering all the variables at each split like in a regular decision tree bagging algorithm. Both RMSE and MAE can be considered very high compared to the median numbers of ARGVs found in each sample, i.e., 2–13 across all cities. In terms of variable importance, the number of ARGs holds the highest predictive value, which matches the high univariate correlation of this feature with the number of ARGVs, followed by bacterial species (top-3 are: *Rahnella* spp. Y9602, *Chlamydia* spp. 2742.308, and *Chlamydia gallinacea*); the first non-species variable is the number of reads, ranked 24th.

### 3.4. Data processing speed

In regards to data processing speed, we compared KARGVA and AMRPlusPlus on a benchmark obtained by clipping a MetaSUB sample (haib17CEM4890_H2NYMCCXY_SL254773) to 12.5, 6.25, and 3.125 million read pairs (approximatively 8, 4, and 2 GB). KARGVA processes on average 1GB of FASTQ data in 00:02:30 hh:mm:ss with processing times of 00:04:31, 00:09:37, and 00:17:52 for, respectively, 2, 4, and 8 GBs inputs. Importantly, KARGVA memory usage is extremely contained, requiring less than 512 MB RAM regardless of the FASTQ size (with 150–300 bp read lengths). We noticed the processing time linearly increasing with file size and the difference between processing compressed vs. uncompressed files is minimal. On the other hand, AMRPlusPlus is about six times slower, with processing times of 00:31:16, 01:01:31, and 01:59:26 for, respectively, 2, 4, and 8 GB inputs.

## 4. Discussion

KARGVA is a fast, multi-platform software for detection of ARGVs from metagenomic high-throughput sequencing experiments. Its ARGV database integrates multiple sources and is the largest available to date, with full linkage to the original sources and their respective ontologies for annotation of resistance class and mechanisms. Our method confirms the detection of resistance mutations, a step that is not included in AMRPlusPlus 2.0 and it is available only for RGI and PointFinder, which however require assembled genomes or genes. KARGVA can thus be used to complement ARG characterization software that does not handle ARGVs, and in particular with KARGA, with which shares strong similarities in the algorithm, command-line options, and output formats.

On semi-synthetic data, KARGVA has high accuracy in presence of non-resistance gene mutations, high error rates, and gene rearrangements. The multiple matching strategy increases ARGV detection accuracy, can report ARGVs that share high genetic similarity, and deal with configurations of resistance mutations not reported in literature. For instance, if a gene G develops resistance either through mutations {A, B, C} or {B, C, D}, and one sample presents with {A, C, D}, its *k*-mers align to G and pass the statistical assessment. However, one possible issue with this approach is that – even if sequences are collapsed – the ARGV database can still store the same ARGV twice or more if there are different laboratory-confirmed configurations of resistance mutations, e.g., using the example above, there would be two entries of for gene G considering both {A, B, C} and {B, C, D} mutations separately.

We also show that KARGVA presents very low false positive rates with respect to bacterial genes not necessarily involved in antimicrobial resistance, as well as specific mutant chromosomal genes that were found in antibiotic-susceptible samples. Although the semi-synthetic data are designed in a rigorous manner, the availability of standardized benchmark datasets from real experiments is auspicated, as discussed by Marini et al. in regards to ARG classifiers (Marini et al., 2022).

A limitation of the software is that the data structures, from a computational point of view, are not memory efficient. While the triple hash table design guarantees most search operations in constant time, there is considerable memory overhead in the padding of Java types/classes and legacy data structures (e.g., the String type and HashMap class). Also, the file parsing/writing is made with a standard BufferedReader and BufferedWriter, simply optimizing the buffer size, and the whole program is implemented serially. The KARGVA database is small, therefore the impact on processing times and memory usage is minimal, and KARGVA is about 6.5 times faster than AMRPlusPlus, independently from the FASTQ file size. Nonetheless, since new antibiotics and new ARGVs are discovered every year, it is advisable to foresee more efficient parsing and *k*-mer handling, considering also succinct data structures and parallelization (Marchet et al., 2020). Also, porting the software to mobile architectures – iOS or Android, and ARM chipsets – is warranted, given the growth of miniaturized, portable, point-of-care sequencing, like Nanopore MinION (Oliva et al., 2020). Since KARGVA is written in Java, the porting to mobile should not be a challenge (although consumer-grade applications have 512 MB or 1 GB memory limit depending on the operating system version), aside needs of optimization, and device overheating issues (Milicchio and Prosperi, 2021). The periodic discovery of new ARGVs not only impacts algorithmic design, but also requires development legacy, by maintaining the curation of data repositories and of software releases. KARGVA utilizes three different sources – CARD, NDARO, MegaRES – that are strongly supported. For instance, MegaRES has been recently updated to v.3.0. with a more advanced characterization of ARGVs (Bonin et al., 2022), including SNP confirmation. The new MegaRES is now being deduplicated and merged with the other sources by the KARGVA developers for future release.

In the re-analysis of the MetaSUB data presented in Danko et al. (2021), we confirm authors’ findings relative to percentages of unclassified reads (41% with Kraken2, in line with our results). We also confirm that – as ARG and ARGV databases grow – there is the expected increase in detection of antimicrobial resistance in the samples. The ARGV profiles retrieved by KARGVA seem more similar across cities than the ARG profiles by AMRPlusPlus. We find a strong correlation between number of ARGs and ARGVs found among samples; however, the median number of retrieved ARGVs is much larger than ARGs, even though ARG databases contain more gene entries. KARGVA substantially improves the ARGV finding rate with respect to other algorithms in the majority of the considered classes.

In accordance with Danko et al. (2021), we find very low concordance between geographic distance among cities and distribution patterns of ARG/ARGVs, Further analysis adding the surface layer does not shed more insights, since there is high heterogeneity in the ARGV city-surface profile pairs. We cautionary do not want to draw any conclusion regarding ARGV patterns among cities, as we expect major unmeasured confounders, and we do not have a reference evolutionary history. The same in fact holds also if analyzing ARGs. To our knowledge, there are no established models to draw evolutionary relationships for metagenomes. Phylogenetic and phylodynamic trees at the species level could be inferred by assembling core genomes, possibly excluding any ARG or resistance mutation to remove bias from convergent evolution, although findings might not be insightful given that samples have been collected at the global level within a small time period –usually such analyses are meaningful for regional outbreaks (Prosperi et al., 2013). Nonetheless, Danko et al. (2021) were able to predict successfully geographic origin of samples using machine learners. Besides city-specific trends in taxa prevalence, ARG/ARGV patterns among cities might be associated to a plethora of factors, from population habits (diet, hygiene), ecological (cleaning schedules of public transportation system, characteristics of the users, e.g., youth, office workers, commuters from rural areas), public health practices (antibiotic usage guidelines and stewardship), in a mixture of common causes, mediators, and common effects for antibiotic resistance.

In conclusion, KARGVA provides reliable characterization of ARGVs, suitable for large metagenomics studies as well as targeted whole genome sequencing, and fills a current toolset and operational gap in a field where only a few limited options are available, with high potential for translational applications.

## Data availability statement

Publicly available datasets were analyzed in this study. MEGARes 2.0 can be found here: https://www.meglab.org/megares/download/. MetaSUB can be found here: http://metasub.org/. KARGVA code can be found here: https://github.com/DataIntellSystLab/KARGVA.

## Author contributions

All authors designed the experiments and wrote the paper. CB and SM developed the computational pipelines. NN provided biological insights and interpretations. MP and SM wrote the KARGVA code and assembled the repository. All authors contributed to the article and approved the submitted version.

## Funding

This work was in part supported by US grants NIH NIAID R01AI141810, NSF SCH 2013998, and USDA AFRI 2019-67017-29110.

## Footnotes

^1^ https://card.mcmaster.ca/

^2^ https://megares.meglab.org/

^3^ https://www.ncbi.nlm.nih.gov/pathogens/antimicrobial-resistance/

^4^ https://github.com/CPTR-ReSeqTB/UVP

^5^ https://www.java.com

^6^ https://github.com/

^7^ https://www.r-project.org/

^8^ https://www.rc.ufl.edu/

## References

[ref1] Alcock B. P. Raphenya A. R. Lau T. T. Y. Tsang K. K. Bouchard M. Edalatmand A.. (2020). CARD 2020: antibiotic resistome surveillance with the comprehensive antibiotic resistance database. Nucleic Acids Res. 48, D517–D525. doi: 10.1093/nar/gkz935, PMID: 31665441 PMC7145624

[ref2] Arango-Argoty G. Garner E. Pruden A. Heath L. S. Vikesland P. Zhang L. (2018). DeepARG: a deep learning approach for predicting antibiotic resistance genes from metagenomic data. Microbiome 6:23. doi: 10.1186/s40168-018-0401-z, PMID: 29391044 PMC5796597

[ref3] Bonin N. Doster E. Worley H. Pinnell L. J. Bravo J. E. Ferm P.. (2022). MEGARes and AMR++, v3. 0: an updated comprehensive database of antimicrobial resistance determinants and an improved software pipeline for classification using high-throughput sequencing. Nucl. Acid Res. 51, D744–D752. doi: 10.1093/nar/gkac1047, PMID: 36382407 PMC9825433

[ref4] Boolchandani M. D’Souza A. W. Dantas G. (2019). Sequencing-based methods and resources to study antimicrobial resistance. Nat. Rev. Genet. 20, 356–370. doi: 10.1038/s41576-019-0108-4, PMID: 30886350 PMC6525649

[ref5] Bortolaia V. Kaas R. S. Ruppe E. Roberts M. C. Schwarz S. Cattoir V.. (2020). ResFinder 4.0 for predictions of phenotypes from genotypes. J. Antimicrob. Chemother. 75, 3491–3500. doi: 10.1093/jac/dkaa345, PMID: 32780112 PMC7662176

[ref6] Coculescu B. (2009). Antimicrobial resistance induced by genetic changes. J. Med. Life 2, 114–123. PMID: 20108530 PMC3018982

[ref7] Danko D. Bezdan D. Afshin E. E. Ahsanuddin S. Bhattacharya C. Butler D. J.. (2021). A global metagenomic map of urban microbiomes and antimicrobial resistance. Cells 184, 3376–3393.e17. doi: 10.1016/j.cell.2021.05.002, PMID: 34043940 PMC8238498

[ref8] Davis J. J. Wattam A. R. Aziz R. K. Brettin T. Butler R. Butler R. M.. (2020). The PATRIC bioinformatics resource center: expanding data and analysis capabilities. Nucleic Acids Res. 48, D606–D612. doi: 10.1093/nar/gkz943, PMID: 31667520 PMC7145515

[ref9] de Abreu V. A. C. Perdigão J. Almeida S. (2021). Metagenomic approaches to analyze antimicrobial resistance: An overview. Front. Genet. 11:575592. doi: 10.3389/fgene.2020.575592, PMID: 33537056 PMC7848172

[ref10] Doster E. Lakin S. M. Dean C. J. Wolfe C. Young J. G. Boucher C.. (2020). MEGARes 2.0: a database for classification of antimicrobial drug, biocide and metal resistance determinants in metagenomic sequence data. Nucleic Acids Res. 48, D561–D569. doi: 10.1093/nar/gkz1010, PMID: 31722416 PMC7145535

[ref11] Gibson M. K. Forsberg K. J. Dantas G. (2015). Improved annotation of antibiotic resistance determinants reveals microbial resistomes cluster by ecology. ISME J. 9, 207–216. doi: 10.1038/ismej.2014.106, PMID: 25003965 PMC4274418

[ref12] Gourlé H. Karlsson-Lindsjö O. Hayer J. Bongcam-Rudloff E. (2019). Simulating Illumina metagenomic data with InSilicoSeq. Bioinformatics 35, 521–522. doi: 10.1093/bioinformatics/bty630, PMID: 30016412 PMC6361232

[ref13] Gu W. Miller S. Chiu C. Y. (2019). Clinical metagenomic next-generation sequencing for pathogen detection. Annu. Rev. Pathol. 14, 319–338. doi: 10.1146/annurev-pathmechdis-012418-012751, PMID: 30355154 PMC6345613

[ref14] Iwu C. D. Korsten L. Okoh A. I. (2020). The incidence of antibiotic resistance within and beyond the agricultural ecosystem: a concern for public health. MicrobiologyOpen 9:e1035. doi: 10.1002/mbo3.1035, PMID: 32710495 PMC7520999

[ref15] Lakin S. M. Kuhnle A. Alipanahi B. Noyes N. R. Dean C. Muggli M.. (2019). Hierarchical hidden Markov models enable accurate and diverse detection of antimicrobial resistance sequences. Commun. Biol. 2:294. doi: 10.1038/s42003-019-0545-9, PMID: 31396574 PMC6684577

[ref16] Marchet C. Boucher C. Puglisi S. J. Medvedev P. Salson M. Chikhi R. (2020). Data structures based on k-mers for querying large collections of sequencing data sets. Genome Res. 31, 1–12. doi: 10.1101/gr.260604.119, PMID: 33328168 PMC7849385

[ref17] Marini S. Mora R. A. Boucher C. Noyes N. R. Prosperi M. (2022). Towards routine employment of computational tools for antimicrobial resistance determination via high-throughput sequencing. Brief. Bioinform. 23:bbac020. doi: 10.1093/bib/bbac020, PMID: 35212354 PMC8921637

[ref18] Marini S. Oliva M. Slizovskiy I. B. das R. Noyes N. R. Kahveci T.. (2022). AMR-meta: a k-mer and metafeature approach to classify antimicrobial resistance from high-throughput short-read metagenomics data. GigaScience 11:giac029. doi: 10.1093/gigascience/giac029, PMID: 35583675 PMC9116207

[ref19] Mason C. Afshinnekoo E. Ahsannudin S. Ghedin E. Read T. Fraser C.. (2016). The metagenomics and Metadesign of the subways and urban biomes (MetaSUB) international consortium inaugural meeting report. Microbiome 4:24. doi: 10.1186/s40168-016-0168-z, PMID: 27255532 PMC4894504

[ref20] Milicchio F. Prosperi M. (2021). “Experimental survey on power dissipation of k-mer-handling data structures for Mobile bioinformatics,” in *2021 IEEE International Conference on Bioinformatics and Biomedicine (BIBM)*, Dec 2021, 3201–3206.

[ref21] Murray C. J. Ikuta K. S. Sharara F. Swetschinski L. Robles Aguilar G. Gray A.. (2022). Global burden of bacterial antimicrobial resistance in 2019: a systematic analysis. Lancet 399, 629–655. doi: 10.1016/S0140-6736(21)02724-0, PMID: 35065702 PMC8841637

[ref22] Nurk S. Meleshko D. Korobeynikov A. Pevzner P. A. (2017). metaSPAdes: a new versatile metagenomic assembler. Genome Res. 27, 824–834. doi: 10.1101/gr.213959.116, PMID: 28298430 PMC5411777

[ref23] O’Leary N. A. Wright M. W. Brister J. R. Ciufo S. Haddad D. McVeigh R.. (2016). Reference sequence (RefSeq) database at NCBI: current status, taxonomic expansion, and functional annotation. Nucleic Acids Res. 44, D733–D745. doi: 10.1093/nar/gkv1189, PMID: 26553804 PMC4702849

[ref24] Oliva M. Milicchio F. King K. Benson G. Boucher C. Prosperi M. (2020). Portable nanopore analytics: are we there yet? Bioinformatics 36, 4399–4405. doi: 10.1093/bioinformatics/btaa237, PMID: 32277811 PMC7828464

[ref25] Prosperi M. Marini S. (2021). KARGA: multi-platform toolkit for k-mer-based antibiotic resistance gene analysis of high-throughput sequencing data. IEEE EMBS Int. Conf. Biomed. Health Informat. 2021, 1–4. doi: 10.1109/BHI50953.2021.9508479, PMID: 34447942 PMC8383893

[ref26] Prosperi M. C. F. Prosperi L. Gray R. R. Salemi M. (2012). On counting the frequency distribution of string motifs in molecular sequences. Int. J. Biomath. 5:1250055. doi: 10.1142/S1793524512500556

[ref27] Prosperi M. Salemi M. Azarian T. Milicchio F. Johnson J. A. Oliva M. (2019). Unexpected predictors of antibiotic resistance in housekeeping genes of staphylococcus aureus. ACM BCB 2019, 259–268. doi: 10.1145/3307339.3342138, PMID: 31592520 PMC6779167

[ref28] Prosperi M. Veras N. Azarian T. Rathore M. Nolan D. Rand K.. (2013). Molecular epidemiology of community-associated methicillin-resistant Staphylococcus aureus in the genomic era: a cross-sectional study. Sci. Rep. 3:1902. doi: 10.1038/srep01902, PMID: 23712667 PMC3664956

[ref29] Sayer E. W. Beck J. Brister J. R. Bolton E. E. Canese K. Comeau D. C.. (2020). Database resources of the National Center for biotechnology information. Nucl. Acids Res. 48, D9–D16. doi: 10.1093/nar/gkz899, PMID: 31602479 PMC6943063

[ref30] Sultan I. Rahman S. Jan A. T. Siddiqui M. T. Mondal A. H. Haq Q. M. R. (2018). Antibiotics, Resistome and resistance mechanisms: a bacterial perspective. Front. Microbiol. 9:2066. doi: 10.3389/fmicb.2018.02066 30298054 PMC6160567

[ref31] Wood D. E. Lu J. Langmead B. (2019). Improved metagenomic analysis with kraken 2. Genome Biol. 20:257. doi: 10.1186/s13059-019-1891-0, PMID: 31779668 PMC6883579

[ref32] Yin X. Jiang X. T. Chai B. Li L. Yang Y. Cole J. R.. (2018). ARGs-OAP v2.0 with an expanded SARG database and hidden Markov models for enhancement characterization and quantification of antibiotic resistance genes in environmental metagenomes. Bioinformatics 34, 2263–2270. doi: 10.1093/bioinformatics/bty053, PMID: 29408954

